# Exploring links between climatic predictability and the evolution of within‐ and transgenerational plasticity

**DOI:** 10.1002/ece3.9662

**Published:** 2022-12-29

**Authors:** Sridhar Halali, Marjo Saastamoinen

**Affiliations:** ^1^ Research Centre for Ecological Change, Faculty of Biological and Environmental Sciences University of Helsinki Helsinki Finland; ^2^ Helsinki Institute of Life Science University of Helsinki Helsinki Finland

**Keywords:** environmental predictability, life history, phenotypic plasticity, time series analysis, (reverse) temperature‐size rule

## Abstract

In variable environments, phenotypic plasticity can increase fitness by providing tight environment‐phenotype matching. However, adaptive plasticity is expected to evolve only when the future selective environment can be predicted based on the prevailing conditions. That is, the juvenile environment should be predictive of the adult environment (within‐generation plasticity) or the parental environment should be predictive of the offspring environment (transgenerational plasticity). Moreover, the environmental predictability can also shape transient responses such as stress response in an adaptive direction. Here, we test links between environmental predictability and the evolution of adaptive plasticity by combining time series analyses and a common garden experiment using temperature as a stressor in a temperate butterfly (*Melitaea cinxia*). Time series analyses revealed that across season fluctuations in temperature over 48 years are overall predictable. However, within the growing season, temperature fluctuations showed high heterogeneity across years with low autocorrelations and the timing of temperature peaks were asynchronous. Most life‐history traits showed strong within‐generation plasticity for temperature and traits such as body size and growth rate broke the temperature‐size rule. Evidence for transgenerational plasticity, however, was weak and detected for only two traits each in an adaptive and non‐adaptive direction. We suggest that the low predictability of temperature fluctuations within the growing season likely disfavors the evolution of adaptive transgenerational plasticity but instead favors strong within‐generation plasticity.

## Graphical abstract

The theory predicts that the evolution of adaptive phenotypic plasticity can only evolve in predictable environments, a core prerequisite that is often overlooked. Here, we test this prediction using environmental time series analyses and a common garden experiment in a univoltine butterfly. In line with the theory, we show that low environmental predictability likely drives the evolution of strong within‐generation plasticity but disfavors adaptive transgenerational plasticity.

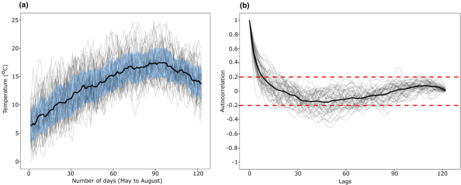

## 1 INTRODUCTION

Phenotypic variation in the wild can be shaped by genetics or by the environment, but often it is an interaction (G × E) of both of these factors. Traditionally, evolutionary biologists have had a strong gene‐centric view in adaptive evolution (Bonduriansky, 2021; Pigliucci, 2005) but the field has witnessed a renaissance in recent years with respect to the contribution of non‐genetic factors at both micro‐ and macroevolutionary scales (Bonduriansky, 2012; Jablonka, 2017; Levis & Pfennig, 2016; Pfennig et al., 2010). One such important non‐genetic phenomenon is transgenerational plasticity (henceforth TGP), where conditions experienced by parents can shape offspring fitness in both adaptive and non‐adaptive manner (Bonduriansky, 2021; Mousseau & Fox, 1998; Uller, 2008). The occurrence of TGP has been widely documented across plants and animals including humans (Uller et al., 2013; Yin et al., 2019), although its adaptive potential has been convincingly demonstrated in relatively few studies (see Agrawal et al., 1999; Fox et al., 1997; Galloway & Etterson, 2007). Detecting adaptive TGP can be difficult and is hindered by factors such as lack of information on species ecology, not using ecologically relevant stressors in the experiments, and meager information on how stressor(s) of interest affects fitness in the wild.

Most organisms live in temporally fluctuating environments, although the degree of fluctuations can differ dramatically across habitats, geographic locations, and so on. When such temporal fluctuations are cyclical, for example, alternating wet‐dry seasons in the tropics or summer‐winters in temperate regions, organisms can make use of the environmental cues (e.g., temperature or photoperiod) to predict the forthcoming selective environment (Chevin & Lande, 2015; Reed et al., 2010). In such predictably fluctuating environments, phenotypic plasticity or within‐generation can be adaptive by providing a tight environment‐phenotype matching using reliable environmental cues (Beldade et al., 2011; Bonamour et al., 2019; Moran, 1992). Seasonal polyphenism in butterflies is a classic example of such adaptive within‐generation plasticity (Shapiro, 1976). Apart from such clear‐cut examples, plasticity can also comprise rapid behavioral and physiological adjustments, for example, as a response to stressful conditions. Even for such transient responses, theory suggests that selection can fine‐tune stress responses when stress‐inducing episodes are predictable (Taborsky et al., 2021).

As the predictability of temporal fluctuations is a pre‐requisite for the evolution of adaptive within‐generation plasticity, similarly, adaptive TGP is expected to evolve when conditions experienced by parents are predictive of the environment experienced by their offspring (Bonduriansky, 2021; Burgess & Marshall, 2014; Leimar & McNamara, 2015; Uller, 2008). In this way, adaptive TGP can provide a jump‐start in enhancing offspring's fitness by shaping their phenotype much earlier during the development (Bell & Hellmann, 2019). Such type of TGP is also called as anticipatory plasticity (Burgess & Marshall, 2014). For example, in *Caenorhabditis elegans*, the evolution of adaptive maternal effects (maternal glycogen provisioning to embryos in anoxic conditions) only occurred when normal and oxygen deprivation conditions fluctuated predictably (Dey et al., 2016, also see Lind et al., 2020). Moreover, when conditions experienced by parents are not predictive of the offspring's environment, TGP can be even maladaptive, and selection is instead expected to favor within‐generation plasticity which may allow a more immediate response to prevailing environmental conditions (Bonduriansky, 2021, Leimar & McNamara, 2015). Despite environmental predictability being a core pre‐requisite in the evolution of both adaptive within‐generation plasticity and TGP (Leimar & McNamara, 2015; Moran, 1992; Reed et al., 2010), thorough analysis of predictability of a relevant stressor(s) using long‐term data remains scant (but see Colicchio & Herman, 2020; Halali et al., 2021). It is even suggested that failure in quantifying the extent of predictability between parent–offspring environments has hindered our understanding of the evolution of adaptive TGP (Burgess & Marshall, 2014; Uller et al., 2013).

Here, by using a combination of environmental time series analyses and a common garden experiment, we investigate how the predictability of the temperature (a stressor in our study) drives within‐generation plasticity and TGP using the Glanville Fritillary butterfly (*Melitaea cinxia*) from the Åland archipelago (south‐west Finland) as a model system. Temperature is an ecologically important stressor and a selective factor that can strongly affect life‐history traits (Atkinson, 1994; Kingsolver & Huey, 2008). It is also a highly relevant and most explored environmental factor in the current scenario of global change (e.g., Ma et al., 2021). Moreover, microclimatic variation in temperature has been shown to have strong effects on key life‐history traits in *M. cinxia* in both field and laboratory settings (Rytteri et al., 2021; Verspagen et al., 2020). Here, we use 48 years (1972–2020) of data on daily temperatures to measure the predictability of temperature fluctuations across and within growing seasons spanning parent and offspring growth periods. We then carry out a common garden experiment to investigate the extent of within‐generation plasticity and the prevalence of adaptive TGP. We expect that treatments with matching parent–offspring temperatures would have higher performance (e.g., higher growth rates) compared to treatments with unmatching temperatures under the evolution of adaptive TGP (for similar findings see Salinas and Munch (2012) and Zizzari and Ellers (2014)). Overall, linking environmental predictability to the results from the experiment allows us to rigorously test the core prediction from the life‐history theory that adaptive TGP is expected to evolve when parental conditions can predict environmental conditions experienced by offspring.

## 2 METHODOLOGY

### 2.1 Study species

*Melitaea cinxia* in the Åland islands inhabits a large network of fragmented dry meadows (called patches) and is one of the classic model systems for metapopulation research (reviewed in Ovaskainen & Saastamoinen, 2018). Finnish *M. cinxia* is univoltine and the life cycle is as follows. Adults emerge in June and females lay eggs in batches on two commonly used hostplants *Plantago lanceolata* and *Veronica spicata* (Wahlberg, 2000). Pre‐diapause larvae (1st to 4/5th instar) hatch in late June/early July and feed gregariously. In late August, larvae spin a communal web (or winter nest) usually comprising of full‐sibs in which they diapause until spring. Post‐diapause larvae (4/5th to 7th instar) recommence their development in early April and move to nearby hostplants. Final instar larvae and pupae in the field are typically found in May (Rytteri et al., 2021).

### 2.2 Quantifying the predictability of temperature fluctuations

The predictability of temperature fluctuations across seasons (i.e., rise and fall of temperature across seasons) and within a growing season was quantified using three different approaches—temporal autocorrelations, wavelet analysis, and Symbolic Aggregation Approximation algorithm. Mean daily air temperature (the average temperature based usually on 4–8 observations per day) available from January 1972 to December 2020 was downloaded from the meteorological station (location: Åland islands, Jomalaby, coordinates: 60.17824, 19.98686; https://en.ilmatieteenlaitos.fi/download‐observations#!/). Temperature values were missing for 22 days across the time series and these gaps were filled with approximated values using ‘*na.approx*’ function from the *Zoo* ver 1.8.9 R package (Zeileis & Grothendieck, 2005). All analyses were performed in R ver 4.1.1 (R Core Team, 2021). R packages *tidyverse* ver 1.3.1 (Wickham et al., 2019) and *ggplot2* ver 3.3.5 (Wickham, 2016) were used for general data handling and producing figures, respectively.

#### 2.2.1 Wavelet analysis

Wavelet analysis or multi‐resolution analysis is a powerful approach that allows quantifying any seasonal phenomenon (Cazelles et al., 2008; Rösch & Schmidbauer, 2018a; Tonkin et al., 2017). Wavelet analysis offers an elegant way to capture both low‐ and (transient) high‐frequency signals in the time series by simultaneously dilating, contracting, and adjusting the height of the mother wavelet (Cazelles et al., 2008, 2014). The dilated wavelet better captures lower frequencies, while the contracted wavelet (simultaneously adjusting its height) better captures high‐frequency signals. Wavelet analysis, therefore, is well suited for detecting abrupt changes in the frequencies such as sharp rise or fall of temperature in the time series (Burgess & Marshall, 2014; Cazelles et al., 2008; Tonkin et al., 2017).

Wavelet analysis was carried out using the *WaveletComp* ver 1.1 (Rösch & Schmidbauer, 2018b) for daily mean temperature time series from 1972 to 2020. The periodicity of the temperature fluctuation across years was visualized using a wavelet spectrum (function: *wt.image*) and the significant periods (*p* < .05 which were determined based on 100 simulations) are indicated by white ridges. Warm colors within the white ridges indicate the regions of the highest power, which represents the magnitude of variance at a given wavelet scale or simply the regions where dominant frequencies oscillate (Rösch & Schmidbauer, 2018a). Moreover, average wavelet power (function: *wt.avg*) was plotted to determine which period of the time series showed the highest power. In addition to the temperature, wavelet analysis was performed on the photoperiod, a perfectly predictable cue, for the above specified years. This allows comparing the extent of predictability in both variables. Time series data for photoperiod for the same location were obtained using the *maptools* package ver 1.1.2 (Bivand & Lewin‐Koh, 2021).

#### 2.2.2 Temporal autocorrelations and symbolic aggregation approximation algorithm

The predictability of temperature within the growing period (April–August) was measured by quantifying temporal autocorrelations and using the Symbolic Aggregation Approximation (SAX) algorithm. Post‐diapause larvae usually start growing from April onwards and the final instar larvae develop during May (Rytteri et al., 2021). Since parental post‐diapause larvae were exposed to temperature treatments in their 6th instar (see next section), temperatures from May to August were used to quantify the degree of predictability separately for all 48 years.

In a time series, temporal autocorrelations measure correlations between the lagged version of itself, which allows identifying until how many days/months the variable (e.g., temperature) is predictable from the starting point. Thus, significant correlations (positive or negative) until the farthest lags will indicate that the environment is highly autocorrelated. Temporal autocorrelations were measured by creating 122 lags (123 total days from May to August) using ‘*tk_acf_diagnostics*’ function from the *timetk* ver 2.6.1 package (Dancho & Vaughan, 2021). It is recommended that the time series should exhibit stationarity while calculating autocorrelations and the raw temperature data did not exhibit stationarity. Thus, residuals obtained by regressing temperature values with the day number separately for each year (e.g., Shama, 2015) were instead used for calculating temporal autocorrelations. Residuals had improved normality (measured using the Shapiro–Wilk test) than the raw values (Figure S1).

Finally, the SAX algorithm was used to determine the predictability of temperature. SAX is an extension of the Piecewise Aggregation Approximation (PAA) algorithm (Lin et al., 2007). PAA algorithm reduces the dimensionality of z‐normalized time series while preserving important information and patterns (Keogh et al., 2001). For example, a time series of length *n* is reduced to any arbitrary length *M* where *M* ≤ *n* but is usually *M* << *n* (Keogh et al., 2001; Lin et al., 2007; see Figure 4a,b). This reduction (*n* to *M*) is carried out by dividing the original time series into *M* frames and then the mean is calculated for each frame. SAX then discretizes PAA data into strings, usually alphabets (Lin et al., 2007, see Figure 4a–c). Thus, by converting continuous time series into strings of alphabets, one could investigate the similarity or repeatability of values in time series across years. First, the original time series (*n* = 120, see below) was reduced to 24 frames (*M*), thus one frame equals 5 days, using the “*paa*” function from the *jmotif* ver 1.1.1 package (Senin, 2020). PAA data were then discretized into seven alphabets from *a*–*g* (i.e., SAX conversion) using “*series_to_chars*” function from *jmotif* package. It is recommended to have an even number of observations in a time series while employing the SAX algorithm (see Lin et al., 2007). Thus, the 31st day of May, July, and August were removed to get an even number of days resulting in *n* = 120 instead of 123 days for each year.

### 2.3 The experiment

Diapausing larvae that were sampled in the autumn of 2020 from the Åland islands were used for the experiment. Larvae were transported to the laboratory and were kept in diapause chambers individually in Eppendorf tubes at 5°C until the start of the experiment in March 2021. Larvae were chosen from five different communes (Finstrom, Hammarland, Jomala, Saltvik, and Sund) having the highest larval abundance (>200) to increase the genetic diversity of individuals in the experiment. When possible, larvae were chosen in such a way as to maximize patch‐level diversity. Larval development was recommenced (*n* = 948) by keeping them at room temperature (~25°C) and spraying them with water. From this point, larvae were monitored for 48 hours and those who failed to show any signs of movement were considered dead. The remaining surviving larvae (*n* = 601) were reared individually in small plastic cups and were fed daily *ad libitum* on fresh leaves of *P. lanceolata*.

On the day of the 5th molt (i.e., mainly 6th instar), the larvae were individually weighed and randomly assigned to three temperature treatments; 28°C, 31°C, and 34°C during the day, and 9°C at night with 12L:12D cycle; in climate‐controlled growth chambers (Sanyo MLR‐350 and a Sanyo MLR‐351). Common hostplants of *M. cinxia* (*P. lanceolata* and *V. spicata*) grow close to the ground and studies show that the ground temperature can be ~10 to 20°C higher than ambient air temperature (Bennett et al., 2015; Rytteri et al., 2021; Singer & Parmesan, 2018). Thus, the temperatures used in our experiment are within the range of temperatures larvae experience in the field at the microhabitat level (see Verspagen et al., 2020). We also acknowledge that photoperiod changes throughout the life cycle of the species (April to August) and thus using constant 12L:12D cycle does not mimic natural conditions. Our rational for omitting manipulation of L:D cycle in the experiment is as follows. First, *M. cinxia* is obligatory univoltine species in Åland, that is, a certain amount of light is needed to break the diapause but the larvae will enter the diapause irrespective of the photoperiod after reaching 4th or 5th instar. This, therefore, indicates that in such obligately univoltine species, expression of life‐history traits is more temperature‐ than photoperiod‐dependent. This argument is further supported by a preliminary experiment with larvae reared at 22 and 28°C at 12L:12D and at 20L:4D, that found temperature to explain most of the variation in diapause strategy (generalized linear mixed model, fixed effect = photoperiod, estimate = −1.021, *p* = .345; fixed effect = temperature, estimate = 4.859, *p* < .001; fixed effect = photoperiod*temperature, estimate = 1.560, *p* = .2; Kahilainen et al., unpublished data, personal communication on 3rd July 2022). Second, simulating changing photoperiod throughout the experiment mimicking natural conditions is logistically challenging, especially for a species such as *M. cinxia* where completing life cycle may take up to 4 months in laboratory conditions. In the 6th instar, ~20% of larval mortality occurred due to parasitoid (*Cotesia* and *Hyposoter*) and some unknown pathogen infestation. Pupae were weighed within 24 hours and upon eclosion, adults were fed on 5% honey water and kept in their respective temperatures before they were assigned for mating. Some mortality also occurred due to failed eclosions. Males from only 28°C treatment were allowed to pair with females from all three temperatures to control for any temperature‐specific paternal effects. The experimental design allows estimating both maternal and paternal effects simultaneously but carrying out an experiment of such a scale was logistically not feasible. Moreover, matings were designed to avoid pair formation between individuals from the same patches (except for one) to reduce the chances of mating between siblings. Mated females were provided with potted *P. lanceolata* plants for laying eggs and were kept in the greenhouse at 28°C.

**FIGURE 1:**
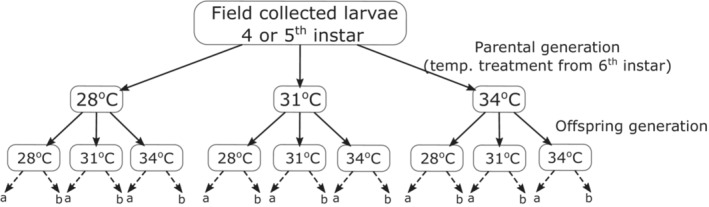
Experimental design used for estimating within‐ and transgenerational plasticity in the Glanville fritillary butterfly (*Melitaea cinxia*). The dashed arrows and letters (a and b) at the bottom of the figure indicate replicate at the plate level (see Section 2).

After laying the first clutch, the total number of eggs were counted, and the clutch was split across three temperature treatments (28°C, 31°C, and 34°C), which resulted in a full factorial split‐brood design where each maternal treatment is divided into three offspring treatments (see Figure 1 for the experimental design). For three females where the number of eggs in the first clutch were low, the second clutch was also split into the same three temperature treatments. Hatched larvae were reared in groups of 15 (larvae of *M. cinxia* are gregarious in the wild) in petri plates lined with the filter paper until diapause. Moreover, a replicate at each plate level was included to account for any plate‐specific effect (Figure 1). During the second molt, three larvae were sampled from each plate for potential genomic analyses in the future. All larvae were fed daily with fresh *P. lanceolata* leaves and sprayed with water to maintain adequate humidity. Some larvae that had extremely long development times and did not show any signs of diapause were culled (*n* = 16).

### 2.4 Measured life‐history traits

*Parental generation*: Post‐diapause larval development time (time taken from the day when larvae entered 6th instar until pupation); pupal weight; growth rate (calculated as: [ln(pupal weight) – ln(6th instar weight)]/larval development time), pupal development time, life‐time fecundity.

*Offspring generation*: egg development time (time taken for eggs to hatch) and hatching success; pre‐diapause larval growth rate (calculated as: ln(larval weight at diapause)/pre‐diapause larval development time; where larval development time is the number of days from hatching until diapause); larval survival (from 1st to 3rd instar & 3rd instar until diapause). Larval survival was measured at two time points as three larvae were sampled at 2nd molt for potential future genomic studies (see above). This approach also allows testing if survival probability differs during early and later stages of larval development.

### 2.5 Statistical analyses

The effect of parental and offspring rearing temperature and their interaction on life‐history traits were tested by fitting linear models (LM), linear mixed effects models (LMM) in the restricted maximum likelihood framework (*lme4* ver 1.1.27.1, Bates et al., 2015) and generalized linear mixed effects models (GLMM) (*glmmTMB* ver 1.1.2.3, Brooks et al., 2017).

*Parental generation*: LMs were fitted to test the effect of temperature, sex, and their interaction on post‐diapause larval development time, pupal weight, growth rate, and pupal development time (all development times were natural log‐transformed). Commune (i.e., location of the larval origin) was not included as a fixed effect as removal of this term considerably improved the model fit (assessed based on the AIC score) and commune level differences in the traits are not of interest in this study. The effect of temperature on fecundity was modeled by fitting a GLMM (family = negative binomial) with clutch order (centered), temperature, and their interaction as fixed effects. Pupal weight (standardized to have a mean of zero and unit standard deviation) was included as a covariate and female id or family as a random effect. Four females that laid just one clutch were removed from the analysis and egg counts up to 11 clutches were included as only a single female laid >12 clutches (16 clutches in total). Note that family could not be included as a random effect for larval traits in the parental generation as these larvae were directly collected from the field, hence, keeping track of the families was not possible.

*Offspring generation*: For egg development time, LMM was fitted with parental‐ and offspring temperature and their interaction as fixed effects, and female id (or family) as a random effect. Similarly, egg‐hatching success was modeled using GLMM with above mentioned fixed effects but specified binomial error structure with a logit link. For larval growth rate and larval survival, LMM and GLMM, respectively, were fitted with parental‐ and offspring temperature, their interaction and plate replicate as an additional fixed effect, and family as a random effect. The number of families across temperatures are as follows: 16 or 17 at 28°C, 14 at 31°C, and 20 at 34°C.

The significance of the fixed effects and their interactions was determined based on the estimate value and their 95% confidence intervals: the effect was deemed significant if the confidence interval did not include zero. Moreover, the significance was also determined using “*Anova*” function from the *car* ver 3.0.11 package (Fox & Weisberg, 2019) and post hoc pairwise contrast between factors using *emmeans* ver 1.7.0 package (Lenth, 2021).

## 3 RESULTS

### 3.1 The predictability of temperature fluctuations across and within seasons

**FIGURE 2:**
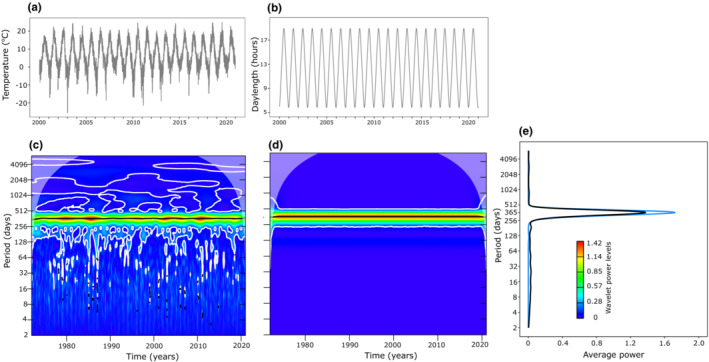
Raw time series of daily mean temperature (a) and photoperiod (b) for Jomalaby location in Åland islands and their wavelet spectra (c and d), respectively, depicting periodicity across the time series. Average power plots (e) for temperature (black curve) and photoperiod (blue curve) indicate the strength of periodicity over a particular period time period (~365 days interval) for both variables. Note that in figures a and b time series data from only 2000 to 2020 is shown for clear visualization, but wavelet analysis was performed on the entire data from 1972 to 2020. In wavelet power spectra plots, the strength of periodicity is indicated by the extent to which warmer colors are distributed over a particular time period. The black solid lines and white contour lines in spectral plots show regions of power significant at the 5% level based on 100 simulations.

**FIGURE 3:**
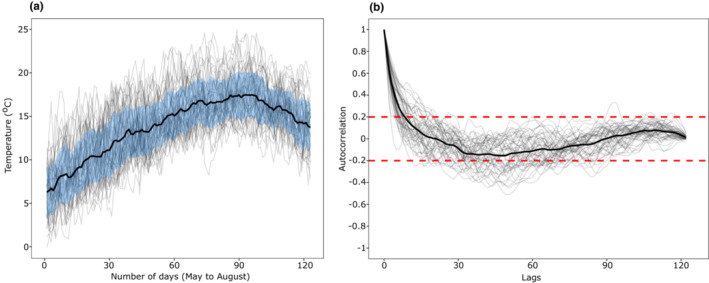
Mean daily temperatures from May–August from 1972 to 2020 (a) and their autocorrelations (b). For both plots, a thin individual line represents a single year and thick black lines indicate the mean temperature (±SD indicated by the blue band) and mean correlation (red lines indicating confidence intervals).

**FIGURE 4:**
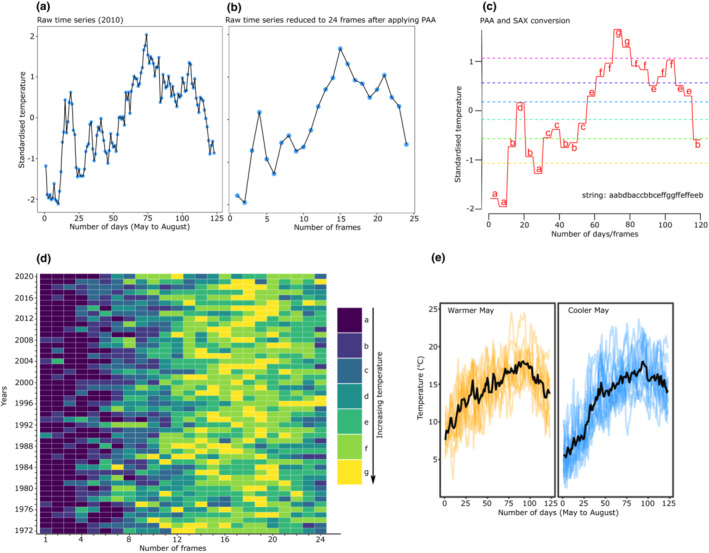
Demonstration of implementation of piecewise aggregation approximation (PAA) and symbolic aggregation approximation (SAX) algorithm on a single year (2010) from May to August (a–c). The raw time series (a, *n* = 123) is reduced to the length (M) of 24 frames after applying the PAA algorithm (b). Figure (c) shows the simultaneous application of PAA and conversion of PAA data into 24‐character string (i.e., SAX conversion). Obtaining such character strings across several years can then be used to investigate the repeatability or predictability of environmental variable(s) across years. The heatmap (d) was obtained after implementing the SAX algorithm for mean daily temperatures from May to August from 1972 to 2020 (here, one frame equals 5 days; see Methods for details). Figure (e) shows that, for a few years which have relatively warmer (orange) and cooler (blue) temperatures in May (mean temperature indicated by thick black lines), does not necessarily translate into warmer or cooler temperatures in June or July.

When compared with the perfectly predictable photoperiod, wavelet spectra for temperature indicated that annual temperature fluctuation is overall predictable (Figure 2). That is, the spectrogram and average power plot for temperature show that there is a dominant frequency recurring at ~365 days intervals (i.e., rise and fall of temperature during summers and winters, respectively) indicated by warm colors (Figure 2). The spectrogram also shows that sharp fluctuations in temperature (indicated by white ridges) are common throughout the time series (Figure 2). Measuring temporal autocorrelations only during the growth period (from May to August) suggests that mean correlation drops rapidly with the first 10 lags and the mean value thereafter remains around zero without exceeding the confidence limits (Figure 3). However, there was high heterogeneity across years with some years showing significant negative correlations between around 30 and 70 lags (Figure 3). Thus, the temperature experienced during May is predictive of temperature during June and early July only during some years (negative correlation indicates higher temperature during June and July compared to May). Finally, comparing patterns of temperature fluctuations across years using the SAX algorithm complements the raw data that temperature generally rises during June and July (Figures 3a and 4). However, the heatmap indicates that the timing of temperature rise is not synchronous across years (Figure 4d). Plotting raw values for a few years showing the highest and lowest mean temperatures during May further suggests that warmer temperatures in spring do not translate into warmer summers and vice versa (Figure 4e).

### 3.2 Within‐generation plasticity: Effect of temperature on parental life‐history traits

**FIGURE 5:**
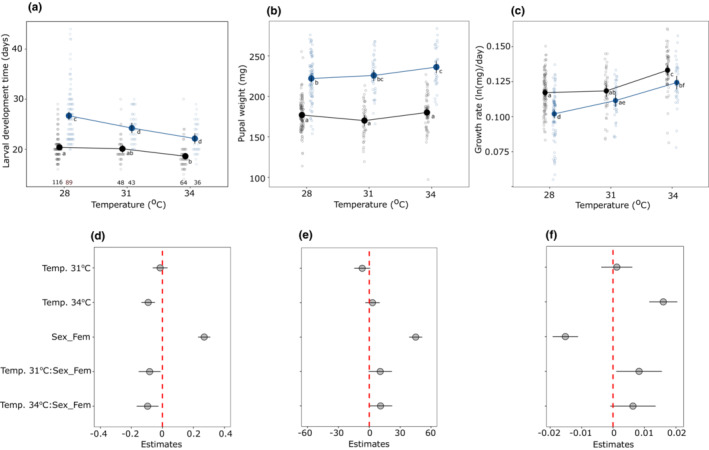
Thermal reaction norms with a model predicted mean (±95% CI) for larval development time (a), pupal weight (b), growth rate (c), and their estimates (±95% CI) for predictors (d–f), respectively. In figures d–f, abbreviations are as follows: Temp. = temperature and sex_Fem = Sex female. In the upper panel, sexes are denoted with different colors (blue = females, black = males) and open circles in the background represent the raw data. Significant differences between groups are indicated by different letters. Sample sizes for males and females, respectively, are provided at the bottom in Figure (a) and these numbers are the same for figures (b) and (c).

**FIGURE 6:**
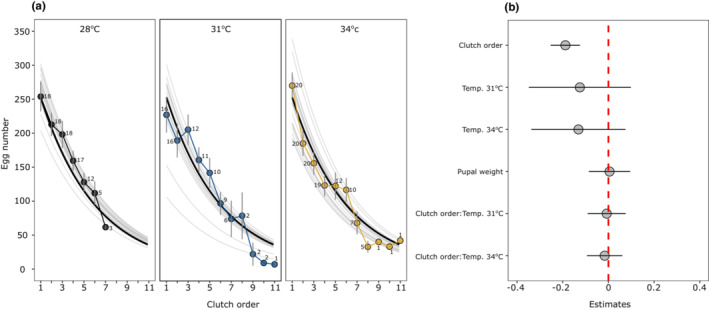
Fecundity curves across temperatures (a) and estimates (±95% CI) for predictors (b). In figure (a), thick and thin black lines show model predicted average egg number and fecundity curves for each female, respectively, and filled circles denote average egg numbers (±SE) from the raw data. Numbers beside each circle indicate the number of females that were laying eggs.

Except for a few traits, rearing temperature during development had a strong effect on life‐history traits (Table S1). Larval development times decreased while pupal weight increased with increasing temperature (Figure 5). Females developed slower and became heavier than males (Figure 5). These translated into the pattern of increasing growth rates with temperature with females having lower growth rates than males (Figure 5). Pupal development time decreased with an increase in temperature without any influence of sex (Figure S2, Table S1). The number of eggs in each clutches decreased substantially with increasing number of clutches without any influence of developmental temperature (Figure 6). Similarly, average clutch size (calculated as the total number of eggs/number of clutches) did not differ across three developmental temperatures (One‐way ANOVA, *F* = 1.18, *df* = 2, *p* = .313, Figure 6). However, there was a trend that females who developed at 28°C laid fewer clutches compared to females who developed at 31°C and 34°C (Figure 6).

### 3.3 Transgenerational plasticity: Effect of parental and offspring rearing temperature on offspring life‐history traits

**FIGURE 7:**
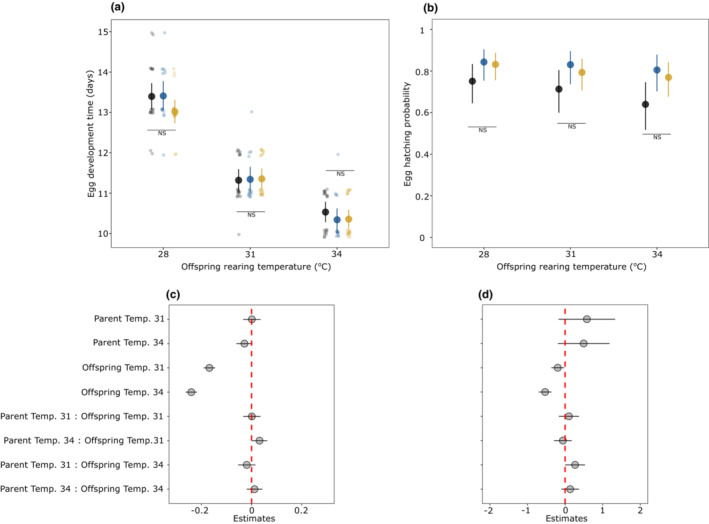
Egg development time with a model predicted mean (±95% CI) (a) and egg‐hatching probability (±95% CI) (b), and estimates of predictors (±95% CI, c and d) for both traits, respectively, across offspring rearing temperatures. In the upper panel, colored filled circles denote parental rearing temperatures (black = 28°C, blue = 31°C, yellow = 34°C). In Figure (a), scattered points in the background show values from the raw data and NS indicates no statistical significance between pairwise contrast across parental rearing temperatures within a single offspring rearing temperature.

**FIGURE 8:**
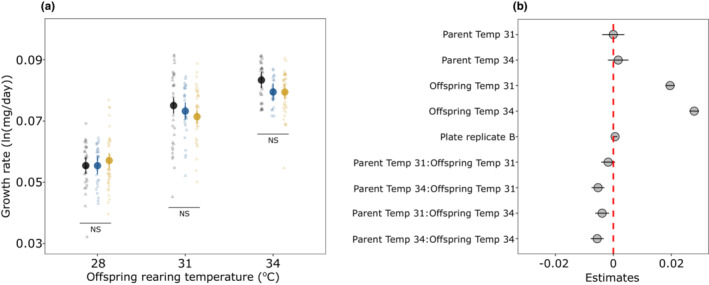
Offspring growth rates with a model predicted mean (±95% CI) across temperatures (a) and estimates of predictors (± 95% CI) (b). In figure (a), colored circles denote parental rearing temperatures (black = 28°C, blue = 31°C, yellow = 34°C), scattered points in the background show values from the raw data with different shapes indicating plate replicates and NS indicates no statistical significance between pairwise contrast across parental rearing temperatures within a single offspring rearing temperature.

**FIGURE 9:**
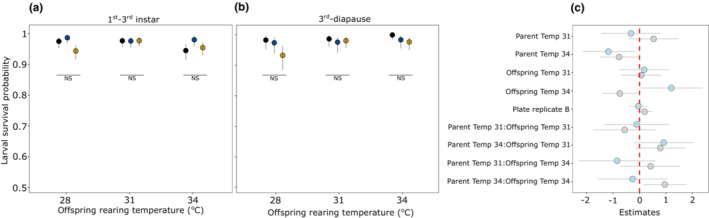
Model predicted larval survival probability (±95% CI) from 1st to 3rd instar (a) and 3rd instar until diapause (b) across offspring rearing temperatures. Figure (c) shows estimates (±95% CI) for predictors with colored circles representing survival at different time points of larval development (gray—1st to 3rd instar, blue—3rd instar until diapause). Colored circles in figures a and b denote parental rearing temperature (black = 28°C, blue = 31°C, yellow = 34°C) and NS indicates no statistical significance between pairwise contrast across parental rearing temperatures within a single offspring rearing temperature.

Under the scenario of adaptive TGP, we expected that treatments with matching parent‐offspring temperatures (e.g., offspring growing at 28°C from parents reared at 28°C) would have higher growth rates than treatments with unmatching temperatures. Egg development time decreased with increasing offspring rearing temperature without any interaction with parental temperature (Parent:Offspring, χ^2^ = 6.57, *df* = 4, *p* = .15, Figure 7a,c, Table S2). Similarly, egg‐hatching success seemed to decrease with increasing offspring temperature without any interaction with parental temperature (Parent:Offspring, χ^2^ = 5.79, *df* = 4, *p* = .21, Figure 7b,d). The post hoc analysis further indicated that (offspring) temperature‐dependent egg‐hatching success was statistically unclear (Figure 7a,b). Larval growth rates increased with increasing offspring temperature but there was also a significant interaction between parent and offspring temperature (Parent:Offspring, χ^2^ = 33.32, *df* = 4, *p* < .001; Figure 8, Table S2). However, the trend was in the opposite direction than expected. That is, at 31° and 34°C offspring rearing temperature, larvae whose parents were reared at 28° had a slightly higher growth rate than those from 31° to 34°, but pairwise comparisons were statistically unclear (Figure 8). Finally, larval survival from 1st to 3rd instar was comparatively lower at 34°C compared to 28° and 31°C offspring rearing temperature (Offspring, χ^2^ = 6.78, *df* = 2, *p* = .03; Figure 9), but there was also a weak interaction with parental temperature (Parent:Offspring, χ^2^ = 11.09, *df* = 4, *p* = .02; Figure 9). More specifically, larvae growing at 34°C from parents reared at 34°C had a slightly higher survival probability than parents reared at 28°C, but not as high as those larvae from parents reared at 31°C (Figure 9). However, this effect was only observed at 34°C offspring temperature with estimates having a relatively wide 95% CI and pairwise comparisons further indicated that this difference was statistically unclear (Figure 9). Such an interaction between parental and offspring temperature was absent when assessing larval survival from 3rd instar until diapause (Parent:Offspring, χ^2^ = 7.09, *df* = 4, *p* = .13, Figure 9, Table S2).

## 4 DISCUSSION

The role phenotypic plasticity, both within‐ and across generations, plays in adaptive evolution has received a renewed interest, even considering us to “rethink” heredity (Bonduriansky, 2012, 2021), and as one of the mechanisms which may allow organisms to cope with climate change (Donelson et al., 2018). Despite its widespread occurrence, renewed appreciation in adaptive evolution, and recent advancements that have unraveled the proximate basis of transgenerational inheritance (e.g., Rechavi et al., 2014), studies linking species ecology and environmental predictability to the evolution of adaptive TGP are rare. Here, using *M. cinxia* as a model system, we show that the larvae exhibit strong within‐generation plasticity, whereas there is only weak evidence of TGP. Furthermore, the evidence of TGP is found for two life‐history traits each in an adaptive and non‐adaptive direction. Our time‐series analyses further showed that, although across‐season temperature fluctuations were fairly predictable, within‐season fluctuations were weakly unpredictable and showed high heterogeneity in predictability (Figure 3). Based on the evidence from both temperature fluctuations and common garden experiment, we posit that our findings, at least to some extent, align with the theoretical prediction that selection will disfavor the evolution of strong adaptive TGP when the predictability of offspring environment is low. We discuss our results in the context of environmental predictability and its role in the evolution of phenotypic plasticity in this butterfly, but it can also be extended to other short‐lived insects in temperate regions.

When parents can predict the offspring conditions based on the prevailing environmental information, adaptive TGP can enhance offspring fitness (Bonduriansky, 2021; Burgess & Marshall, 2014). However, environmental predictability is scarcely quantified despite being a core prerequisite for the evolution of adaptive TGP, especially in non‐model organisms (but see Burgess & Marshall, 2011; Diaz et al., 2021; Halali et al., 2021; Shama, 2015). Experimental evolution studies on species with short generation times have explicitly shown that adaptive TGP readily evolves when selection lines are maintained in a temporally autocorrelated environment (e.g., Dey et al., 2016; Lind et al., 2020; Rescan et al., 2020). Few empirical studies that have quantified the predictability between parent–offspring environments have measured autocorrelations on a shorter time series (usually a few months within a year), which precludes accounting for climatic heterogeneity across years. Moreover, temporal autocorrelations suffer from the drawback that sample size decreases substantially with increasing lags and correlations for farthest lags can be unreliable. Other much informative and powerful methods such as Fourier and wavelet transform allow quantifying environmental predictability (e.g., Burgess & Marshall, 2014; Halali et al., 2021; Marshall & Burgess, 2015; Tonkin et al., 2017) but are seldom used. Here, we use wavelet analyses, temporal autocorrelation, and the SAX algorithm to quantify the predictability of temperature fluctuations across and within the growing season as the degree of predictability at different scales can have different evolutionary implications for the evolution of phenotypic plasticity.

One striking pattern that was observed was that the species followed the reverse temperature‐size rule. The reverse temperature‐size rule is the opposite of the more widespread pattern observed in ectotherms: the temperature‐size rule (TSR) where body size decreases with increasing temperature (Atkinson, 1994). Although whether TSR or reverse‐TSR is adaptive remains debated (Atkinson & Sibly, 1997), some studies have shown that factors such as temperature (Huey et al., 2000; Kingsolver et al., 2007) and food quality (Diamond & Kingsolver, 2010) can readily mold the slope of the thermal reaction norm. The length of the season available for breeding (or season length) is another prominent selective factor which is expected to result in reverse‐TSR (Blanckenhorn & Demont, 2004; Mousseau, 1997). Season length decreases considerably with increasing latitude. Thus, ectothermic species or populations at higher latitudes are predicted to achieve larger size at maturity by evolving genetically faster growth rates to compensate for seasonal time constraints, a hypothesis called as countergradient variation (Blanckenhorn & Demont, 2004). While life‐history theory posits that juvenile development time is positively correlated with body size at maturity (Nylin & Gotthard, 1998), under countergradient hypothesis, there is decoupling between these two key life‐history traits. It should be noted that countergradient variation hypothesis is used for explaining clinal variation in body size across populations or species (see Blanckenhorn & Demont, 2004). While our study was based on single population of *M. cinxia* at northern latitude (60°N), the countergradient variation hypothesis may still allow explaining within‐population pattern of reverse‐TSR. We speculate that since selection can readily decouple association between juvenile (larval) development and body size at higher latitudes, this allows individuals to achieve larger body size by increasing growth rates and reducing development time at higher temperatures. In the field where temperature variation within the season is unpredictable (Figure 3), such decoupling may allow individuals to achieve higher growth rates on warm days without compensating for the body size. Larger body size is associated with increased fitness, for example, by increasing fecundity (Honěk, 1993). Interestingly, at higher temperatures where females had larger body size (pupal weight as a proxy), we did not find any strong effect on fecundity (Figure 6), but this correlation was apparent in another study where fecundity was assessed under semi‐natural field conditions (see Rosa & Saastamoinen, 2017). Overall, we posit that reverse‐TSR is likely adaptive in high‐latitude population of *M. cinxia* (and likely in other high‐latitude univoltine species) shaped by complex interaction between seasonal time constraints and within‐season temperature variation.

Fine‐scale variation in temperature and its predictability within the growing season can have important consequences for the evolution of TGP. Temporal autocorrelations show that average correlations are near zero suggesting temperatures during May do not or are extremely weakly predictive of temperatures in succeeding months (Figure 3). Moreover, the heatmap derived from the SAX algorithm (Figure 4d) suggests that, as expected, June and July are relatively warmer, but the timing of temperature peaks is not synchronous across years. Examining raw temperatures further show that years having relatively warmer temperatures during May does not necessarily translate into a warmer summer (i.e., June and July, Figure 4e). This, therefore, indicates that temperatures experienced by parents during their growth are not or are only weakly predictive of the temperature that will be experienced by the offspring. Overall, we posit that evidence of weak adaptive TGP in our system may have been due to lower predictability of temperature within the growing season and selection may have instead favored the evolution of strong within‐generation plasticity which provides a more rapid response to the prevailing conditions.

Interestingly, there was an interaction between parental and offspring temperature for two offspring life‐history traits (pre‐diapause larval growth rate and survival), but both had low effect sizes with relatively wide confidence intervals. We found that larvae whose mothers were reared at 28°C had slightly higher growth rates at warmer, 31°C and 34°C, offspring rearing temperatures than larvae from mothers reared at warmer temperatures (Figure 8). This pattern is, however, in the opposite direction than we initially expected (i.e., that offspring's growing in similar temperatures as that of their parents would have higher performance). We speculate that this trend may still be adaptive, as 28°C is not optimal for achieving high growth rates, and thus parents who developed at 28°C may prime their offspring to attain higher growth rates in warmer conditions. Furthermore, there was weak evidence for TGP toward the expected direction for offspring survival from 1st to 3rd instar. That is, at 34°C offspring rearing temperature, offsprings whose parents were reared at higher temperature (31° & 34°C) had slightly higher survival (Figure 9a). However, this weak effect was only observed at 34°C offspring rearing temperature and this trend was absent when larval survival was measured from the 3rd instar until diapause. Moreover, there was an indication that, especially when estimating fitness in terms of offspring survival, 34°C appeared to be a stressful condition. Theoretically, it is argued that TGP is not always adaptive, in fact, in most instances, it is expected to be mal‐ or non‐adaptive (Bonduriansky, 2021). TGP may occur from factors other than adaptive reasons such as high physiological sensitivity to environmental fluctuations during reproduction or physiological constraints (Bonduriansky, 2021). Significant parent–offspring interactions for two life‐history traits in this study and evidence from other studies in *M. cinxia* (Saastamoinen, Hirai, & van Nouhuys, 2013; Salgado & Saastamoinen, 2019) do suggest that TGP is prevalent in this system. However, whether the TGP is actually adaptive will require new ways of measuring fitness in both the laboratory and the wild.

Commonly used hostplants of *M. cinxia*, *Plantago lanceolata*, and *Veronica spicata*, grow close to the ground and studies have shown that ground temperature can be ~10–20°C degrees warmer than ambient air temperature (Bennett et al., 2015; Singer & Parmesan, 2018). First, we acknowledge that using time series data on the ground temperature in microhabitats of *M. cinxia* would have been ideal, but such data are not yet available. However, studies have suggested that there is generally a strong correlation between air and ground temperature (Tsilingiridis & Papakostas, 2014). Moreover, during exceptionally warm years such as in 2018 when summer temperatures were >25° in Åland (see van Bergen et al., 2020), ground temperatures may have easily exceeded 34°C which was the warmest treatment in our experiment. Future studies using higher stress‐inducing temperatures in the experiments will be important in investigating the prevalence of adaptive TGP. Second, the constant 12L:12D conditions used in our experiment do not mimic natural conditions, especially as the photoperiod changes throughout the life cycle of the species from April to August. However, as argued before (see Methodology section), given the species is obligately univoltine, expression of life‐history traits is likely to be more temperature‐dependent than photoperiod. Thus, we believe that the findings of our study will hold even if different light conditions are used. Moreover, simulating changing photoperiod mimicking natural conditions is logistically challenging. Finally, we also acknowledge that, since field‐caught larvae were directly used in the experiment, our study is not able to control for the conditions that parents may have experienced at the pre‐diapause stage (i.e., from egg to 5th instar). Given that mortality occurred at the parental stage, there is a possibility that parents may have experienced selection and mortality may have weeded out some genetic background capable of expressing adaptive TGP. We, thus, purposely chose larvae from diverse localities (or communes) across Åland to increase the genetic diversity and included a large number of families across temperatures in the experiment. We recognize these caveats and one way of controlling this noise would have been to rear at least one complete generation in the laboratory before starting the experiment. However, working with univoltine systems such as *M. cinxia* in a laboratory setting poses substantial logistical challenges and larval mortality, especially during diapause, is common even in the best possible artificial conditions. Despite the shortcomings, we believe our study establishes a clear link between seasonal predictability and the evolution of adaptive within‐ and transgenerational plasticity in line with the theoretical prediction.

One of the notable effects of climate change, especially at the higher latitudes, is that the springs are arriving early, and summers are getting longer (Bradshaw & Holzapfel, 2001). Climate change is also predicted to increase temporal and spatial autocorrelation of temperature in temperate regions (Di Cecco & Gouhier, 2018; Kahilainen et al., 2018). Studies in both plants and animals have indicated that TGP might be one of the mechanisms enabling species to buffer the effects of climate change (e.g., Groot et al., 2017). Given that we find very weak evidence for adaptive TGP, it will be of great interest to investigate if the species could evolve such a response on a contemporary time scale. Future studies using simulated climate change experiments (e.g., Shama et al., 2014) would allow testing such a hypothesis. Moreover, performing experiments similar to the current study on *M. cinxia* populations from lower latitudes (e.g., from France, Spain, Morocco; similar to Munch et al., 2021), where temporal autocorrelations for temperature are expected to be higher would allow investigating how climatic predictability drives the evolution adaptive TGP.

## AUTHOR CONTRIBUTIONS

**Sridhar Halali:** Conceptualization (lead); data curation (lead); formal analysis (lead); investigation (lead); methodology (equal); project administration (lead); supervision (equal); validation (equal); visualization (lead); writing – original draft (lead); writing – review and editing (lead). **Marjo Saastamoinen:** Conceptualization (equal); formal analysis (supporting); funding acquisition (lead); methodology (equal); project administration (supporting); resources (lead); supervision (equal); validation (equal); writing – original draft (supporting); writing – review and editing (supporting).

## CONFLICT OF INTEREST

The authors have no conflict of interest to declare.

## Supporting information


Appendix S1

## Data Availability

All data files are available in the Dryad repository: https://doi.org/10.5061/dryad.9ghx3ffmz.
